# Karst grassland forage quality and its determinants in Guizhou Province of Southwest China

**DOI:** 10.7717/peerj.15323

**Published:** 2023-05-16

**Authors:** Dengming He, Baocheng Jin, Xuechun Zhao, Hua Cheng, Chao Chen, Huanhuan Wang, Jinping Zhang, Yaoyao Zhang, Qin Yang, Kun Liu, Min Han, Zhongcai Li, Jing Peng

**Affiliations:** 1 College of Animal Science, Guizhou University, Guiyang, Guizhou, China; 2 School of Tourism, Henan Normal University, Xinxiang, Henan, China; 3 Guizhou Institute of Natural Resources Survey and Planning, Guizhou Department of Natural Resources, Guiyang, Guizhou, China

**Keywords:** Biodiversity, Climate change, Grazing, Nutrient-dilution effect, Karst

## Abstract

Forage quality is a key property of grassland ecosystems. In this study, grassland forage qualities were measured at 373 sampling sites throughout Guizhou Province in the karst mountain region of Southwest China, and the factors affecting it were explored. The forage quality level of most plant species was categorized into four levels: (1) preferred forage species; (2) desirable forage species; (3) consumed but undesirable forage species; and (4) non-consumable or toxic forage species. High temperature and precipitation appeared to facilitate the growth of preferred forage species, but limited the growth of other plants. Increasing soil pH had a positive impact on the number and biomass of preferred forage plants, but a negative influence on other plants, especially non-consumable or toxic plants. Both GDP and population density had a positive correlation with the number and biomass of preferred forage species, while such correlations for other levels of forage species tended to be negative. Grazing could lead to a decrease in the preferred forage species. Therefore, it is suggested that by focusing on soil improvement in grassland and maintaining an appropriate grazing intensity, global warming and rapid economic growth in Guizhou Province will likely contribute to increase the forage quality of karst grasslands in Southwest China.

## Introduction

Grassland ecosystems cover about 25% of the global land surface (Asner et al., 2004; Gong et al., 2013; Yu, Wang & Gong, 2013; Bengtsson et al., 2019) and play an important role in livestock, dairy, and meat production and in the livelihoods of millions of local farmers (Kemp & Michalk, 2007; Godde et al., 2020; Zhao, Liu & Wu, 2020). Grassland forage quality is a key property of grassland ecosystems (Vander Wal et al., 2000), and it influences both plant digestibility and livestock performance (Bokdam & De Vries Wallis, 1992; Kawamura et al., 2008; Shi et al., 2013). Grassland quality can be affected by various factors, including climate (Klein, Harte & Zhao, 2007; Walter et al., 2012; Jentsch et al., 2014; Dumont et al., 2015; Xu et al., 2018; Carni et al., 2021; Seibert et al., 2021), species composition (Michaud et al., 2015; Andueza et al., 2016; Bernardi, Jonge & Holmgren, 2016; French, 2017; Schaub et al., 2020), grazing pressure (Anderson et al., 2007; Schonbach et al., 2009; Zhai et al., 2018), and soil properties (Petit Hélène et al., 1992; Perotti et al., 2021).

There has been some disagreement regarding the implications of climate factors on grassland quality. Some studies have found that warming can increase forage production by facilitating plant growth (Lin, Xia & Wan, 2010; Bai et al., 2013; Polley et al., 2013), while it has also been shown that warming could decrease forage quality *via* nutrient-dilution effects (Shi et al., 2013; Chen et al., 2020). Later investigations have shown that warming is projected to alter plant community compositions and increase rangeland grass quality in the Tibetan Plateau (Wang et al., 2012; Li et al., 2018a, Li et al., 2018b; Li et al., 2018c). However, such research has not focused on the karst mountain region of Southwest China.

Previous research has also shown that soil attributes (including soil bulk density, soil organic carbon, soil total nitrogen, soil total phosphorus, and soil available phosphorus) were not important factors affecting grassland quality across Chinese grasslands at a large scale (Shi et al., 2013). However, this previous study has failed to consider soil pH, which is a very important soil attribute, especially in the karst mountain region of Guizhou Province, Southwest China (Liang et al., 2016; Xiao et al., 2020), where more than 80% (83.7%) of the area is characterized by acidic soil (pH <7.0) due to its high precipitation than evapotranspiration and local soil types (FAO et al., 2012; Slessarev et al., 2016; Chen et al., 2019). Karst areas, characterized by the presence of karstifiable carbonate rock, represent approximately 15% of the world’s terrestrial zones, and almost 17% of the human population lives there (Goldscheider et al., 2020; Canedoli et al., 2022). In addition, the karst region of Southwest China is one of the largest continuous karsts in the world and has unique landscapes and fragile ecosystems (Huang, Cai & Xing, 2008; Jiang, Lian & Qin, 2014; Gao et al., 2021). Grasslands are important for the composition of local karst ecosystems and crucial for the livelihoods of local farmers, ecological restoration, and sustainable development (Yang et al., 2022). There is no previous literature available that has assessed the quality of grassland forage and the factors affecting its distribution in the karst mountain region of Guizhou Province in Southwest China or in similar areas.

For the human activities factor, previous research in China has found that better economic conditions will likely result in greater investment for grassland improvement (Shang et al., 2014; Briske et al., 2015). In recent years, the rapid economic growth in Guizhou Province and other provinces in the karst mountain region of Southwest China (National Bureau of Statistics of China, https://data.stats.gov.cn/english/, accessed on August 15, 2021) may facilitate grassland quality improvement.

Previous studies have also shown that heavy grazing results in the rapid consumption of high-quality forage species, thus reducing the forage quality of grasslands (Bokdam & De Vries Wallis, 1992; Asner et al., 2004). However, at the same time, several studies have also shown that short-term or light grazing pressure is unlikely to cause grassland degradation and decrease in grassland quality (Milchunas & Lauenroth, 1993; Klein, Harte & Zhao, 2007; Anderson et al., 2007; Schonbach et al., 2009; Mbatha & Ward, 2010; Li et al., 2018a, Li et al., 2018b; Li et al., 2018c).

The present study was conducted to achieve the following objectives: (1) to characterize the spatial distribution of grassland forage quality in the karst mountain region of Guizhou Province in Southwest China by categorizing the forage quality of each grassland plant species into four levels including preferred forage species, desirable forage species, consumed but undesirable forage species, and non-consumable or toxic forage species (2) to assess the factors affecting the grassland quality spatial distribution pattern, and (3) to explore appropriate management strategies to improve grassland quality in karst mountain regions.

## Materials & Methods

**Figure 1:**
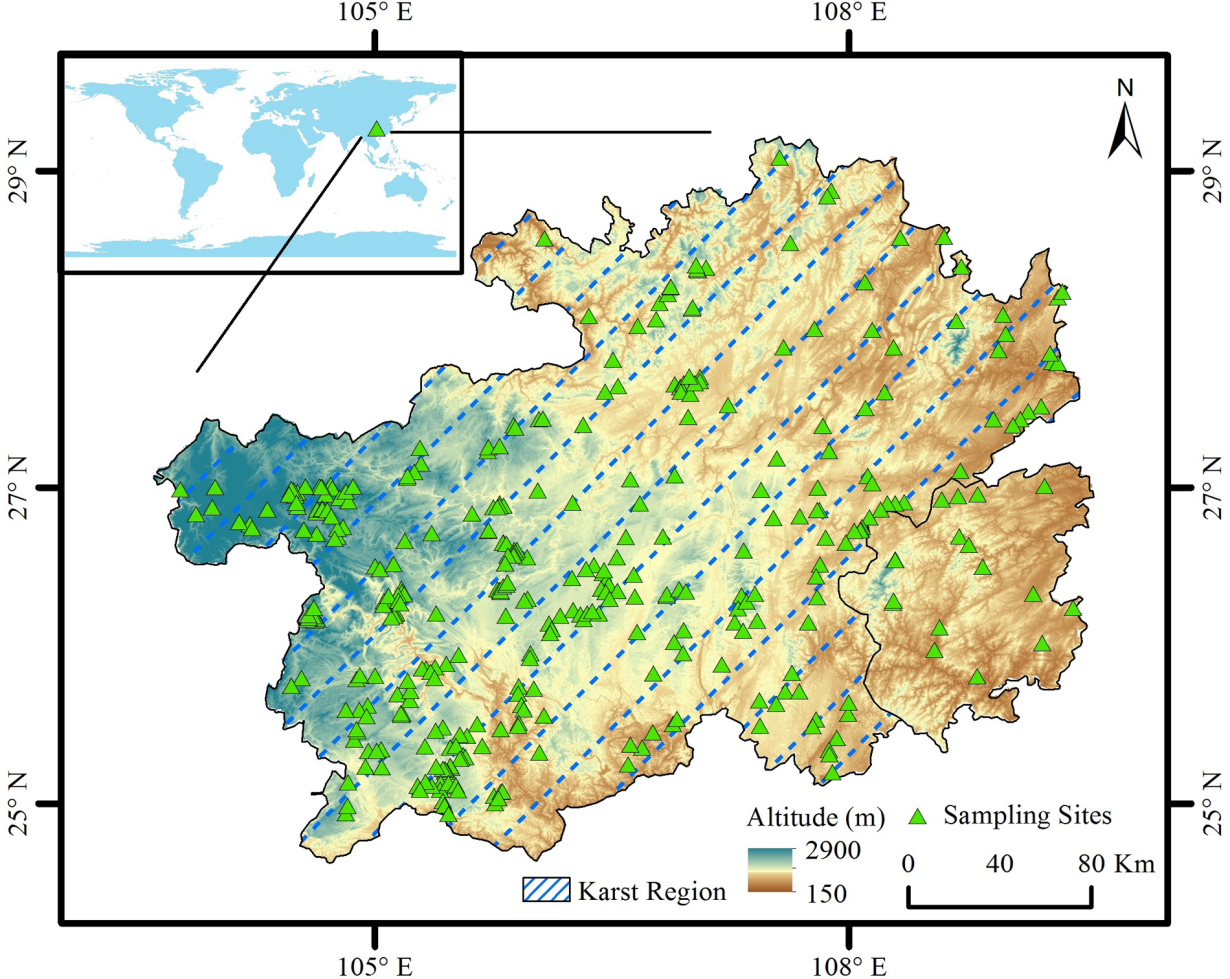
Distribution of sampling sites in Guizhou Province, Southwest China. The boundary of karst region in Guizhou Province was derived from Wang et al. (2019) and Ford & Williams (2007).

The present research was conducted in the karst mountain region of Guizhou Province in Southwest China (24°37′–29°13′ N, 103°36′–109°35′ E; 150–2,900 m elevation; Fig. 1). The region has a humid subtropical monsoon climate. The mean (1981–2010) annual temperature is 14.2 °C, and the mean annual precipitation is 1,069.9 mm. For the coldest (January) and warmest (July) months, the daily temperatures were 4.4 °C and 22.2 °C, respectively (data from the China Meteorological Data Center; https://data.cma.cn/, accessed on August 15, 2021, Fig. 2). Grassland is one of the most important ecosystem types in Guizhou Province, as grasslands (including natural grasslands, artificial grasslands, and abandoned fields) cover more than 200,000 hectares of the province (data from the third national land survey of China, see Chen et al., 2022). The grasslands are commonly used for livestock grazing, cutting forage, and rural tourism. The common livestock is cattle, horse, sheep, and goat. The main soil types in the region are Haplic Alisols, Haplic Luvisols, Dystric Regosols (FAO 90 taxonomy), and acidic soil (pH < 7.0), which accounts for about 83.7% of the land surface of the province (FAO et al., 2012). The region is covered by karst mountain terrain. The grassland plant community is mainly composed of *Imperata cylindrica* (L.) P. Beauv, *Miscanthus floridulus* (Labill.) Warb. ex K. Schum. & Lauterb, *Pennisetum sinese* Roxb, *Eragrostis pilosa* (L.) P. Beauv., *Arundinella hirta* (Thunb.) Tanaka, *Heteropogon contortus* (L.) P. Beauv. ex Roem. & Schult., and *Macrothelypteris oligophlebia* (Baker) Ching.

**Figure 2:**
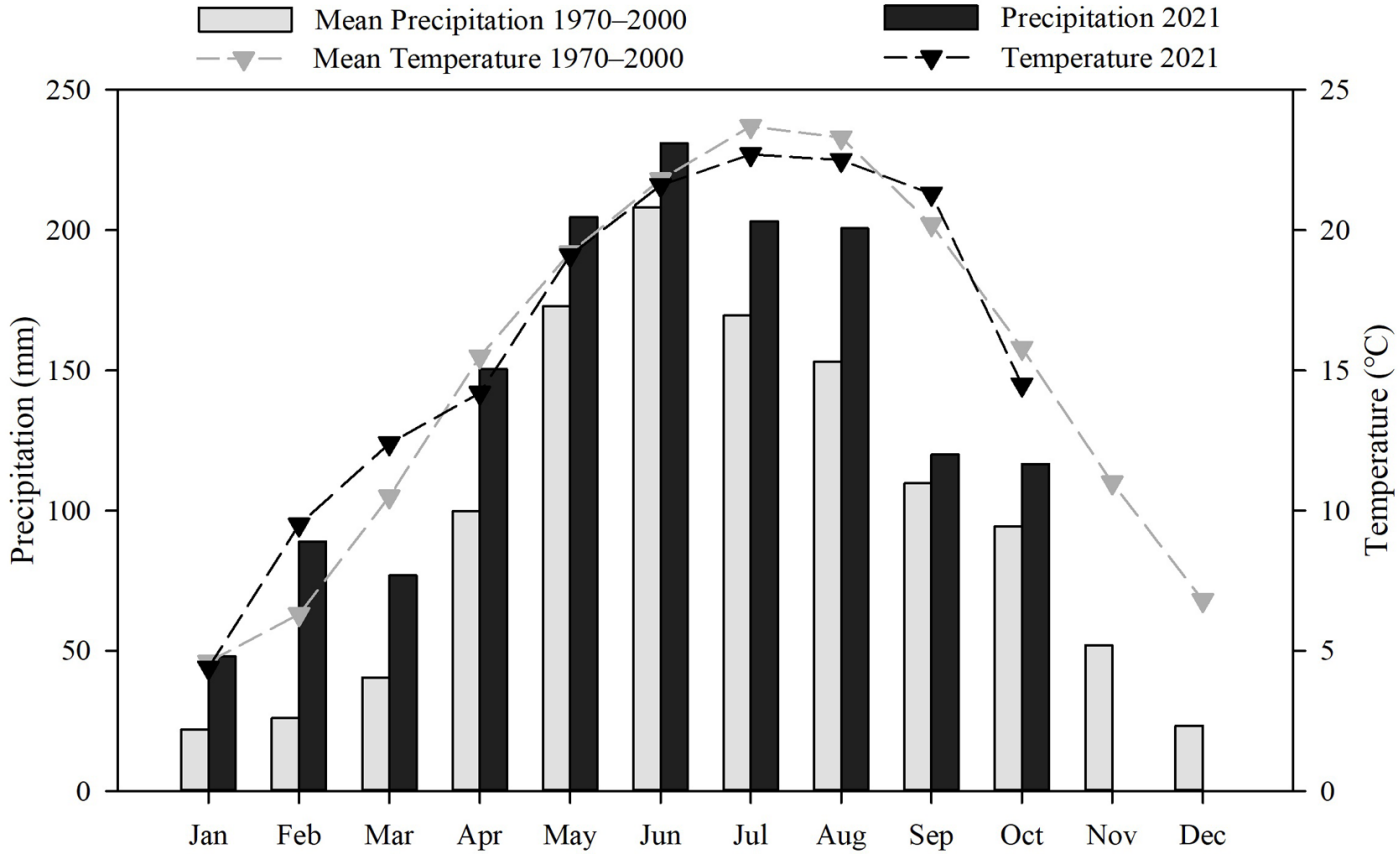
Mean monthly precipitation and temperature patterns in Guizhou Province, China.

Overall, 373 sampling sites were selected throughout Guizhou Province in Southwest China from July to October, 2021 under the approval from Guizhou Department of Natural Resources. Over 95% (356/373) of the sampling sites were located in the karst mountain region (Fig. 1). Within each sampling site, three 1 m ×1 m sampling plots were established using a 1 m ×1 m sampling frame. The three sampling plots were well separated from each other with a minimal distancing of about 20 m. Within each sampling plot, the numbers of species, average plant height, plant cover, and plant biomass were determined. The plant height (natural position) was acquired using a measuring tape. The plant cover was acquired by taking photos (photo line transect method, see Wang et al., 2022). The plants in each plot were collected with ground level stubble height and separated by species. Vegetation samples were dried for 90 h at a temperature of 60 °C and weighed to determine the aboveground plant biomass. The grazing pressure within each site was classified into no grazing and grazing, based on field measurements of livestock excrement, trampling routes (Jin et al., 2016; Jin et al., 2019), and interviews of local residents.

The quality level of each forage species was determined according to the publication “China Forage Plants” (Chen & Jia, 2002), the online database “Scientific Database of China Plant Species” (http://db.kib.ac.cn/, accessed on March 21, 2022), and other related references (Damiran, 2005; Ye & Du, 2014; Huang et al., 2018; Zhao et al., 2020). The forage species were categorized into the following four levels: (1) preferred forage species, these species were preferred by livestock and consumed far in excess of its vegetative composition; (2) desirable forage species, readily eaten but corresponding to a lesser portion than preferred plants; (3) consumed but undesirable forage species, eaten by livestock but usually comprising a minor part of the diet or consumed in a much smaller proportion relative to the vegetative composition; (4) non-consumable or toxic forage species, not eaten by livestock intentionally or containing toxic substances (Damiran, 2005). The forage quality levels are referring based on the consumption of main types of local livestock including cattle, horses, sheep, and goats.

The corresponding social and environmental factors were also recorded throughout the study area, including elevation (m), mean annual temperature (1970–2000, °C), mean annual precipitation (1970–2000, mm) (Fick & Hijmans, 2017), soil bulk density (SBD, 0–30 cm), soil organic carbon (SOC, 0–30 cm), and soil pH (0–30 cm) (FAO et al., 2012), GDP (Gross Domestic Product, average of 2000, 2005, 2010, 2015, and 2019), and population density (average of 2000, 2005, 2010, 2015, and 2019) (Yi, Xiong & Yang, 2006; Huang et al., 2009; Han et al., 2011; Xu, 2017) (Table 1). The soil data was derived from harmonized world soil database (version 1.2) (FAO et al., 2012). The GDP and population density data was derived form 1 km grid GDP and population density data of China, and the data of each sampling site indicated the GDP and population density of its corresponding 1 km ×1 km grid. The 1 km grid GDP and population density data of China were derived from spatial interpolation based on county level data. The mean monthly temperature and total precipitation (January–October) for the sampling year 2021 were derived from ERA5-Land monthly averaged data (Muñoz Sabater, 2019; Muñoz Sabater, 2021). The reanalysis 2021 climate data, with a spatial resolution of 0.25° ×0.25° , combines model data with observations from all over the world. The correlation coefficients of grassland quality were calculated using IBM SPSS Statistics (version 19, IBM Corp., Armonk, NY, USA). One-way ANOVA was used to test the differences between no grazing and grazing sampling sites for the relevant variables. The normality of each variable was tested in R using the Shapiro–Wilk analysis (version 3.6.3, R Core Team, 2020).

**Table 1:** Possible social and environmental factors affecting grassland quality.

Item	Source
Elevation	ASTER GDEM (https://www.gscloud.cn/, accessed on March 21, 2022)
Mean annual temperature	WorldClim Data version 2.1 (https://www.worldclim.org/, accessed on March 21, 2022)
Mean annual precipitation	
Soil bulk density (SBD)	Harmonized World Soil Database version 1.21 (http://webarchive.iiasa.ac.at/Research/LUC/External-World-soil-database/, accessed on March 21, 2022)
Soil organic carbon (SOC)	
pH	
Gross domestic product (GDP)	Resource and Environment Science and Data Center (http://www.resdc.cn/, accessed on March 21, 2022)
Population density	

## Results

The results of the study show that 483 forage species were found within 373 sampling sites, among which the forage quality levels of 272 species belonging to 48 families and 177 genera were measured (for the forage quality level of each plant species, see Appendix Table S1). These 272 species accounted for 85.9% of the biomass of all the 483 species. There were 65 preferred forage species, 151 desirable forage species, 33 consumed but undesirable forage species, and 23 non-consumable or toxic forage species.

The main families of 65 preferred forage species were Poaceae (27 species), Fabaceae (15 species), and Asteraceae (five species). The main families of 151 desirable forage species were Poaceae (31 species), Asteraceae (27 species), Fabaceae (25 species), Rosaceae (nine species), Cyperaceae (eight species), Polygonaceae (five species), and Gentianaceae (five species). The main families of 33 consumed but undesirable forage species were Asteraceae (20 species), Lamiaceae (three species), Poaceae (two species), and Caprifoliaceae (two species). The main families of 23 non-consumable or toxic forage species were Pteridaceae (five species), Asteraceae (three species), Thelypteridaceae (three species), and Blechnaceae (two species).

There were significant positive correlations between the number of forage species of all four forage quality levels and elevation (Table 2). For number, percentage, and biomass (biomass and biomass percentage), the correlations were significantly negative for preferred forage species (level 1), but positive for other forage species (Table 2). For both precipitation and temperature (including mean annual temperature, mean annual precipitation, and precipitation and temperature in the year 2021), the correlations between the number and biomass of preferred forage species were positive, while such correlations were negative for the other types of forage species. The correlations between SBD and SOC with the occurrence of various types of forage species were mostly non-significant. There were negative correlations observed between soil pH and the number of forage species of all forage quality levels. For forage species number, percentage, and biomass (biomass and its percentage), the correlation tended to be positive for preferred forage species, but negative for consumed but undesirable and non-consumable or toxic forage species (Table 2). For both GDP and population density, there tended to be positive correlations with the number percentage and biomass of preferred forage species, however, there was a negative correlation with the number of preferred forage species. The correlations for other forage species were negative (Table 2).

**Table 2:** Spearman’s correction coefficients between grassland quality and social and environmental factors.

	**Forage Quality Level**	**Elevation**	**Precipitation Mean** **1970–2000**	**Temperature Mean** **1970–2000**	**Precipitation 2021**	**Temperature 2021**	**SBD**	**SOC**	**pH**	**GDP**	**Population Density**
Number of Forage Species	1	0.24^*^	0.06	−0.21^*^	0.13^*^	−0.12^*^	−0.08	−0.04	−0.12^*^	−0.11^*^	−0.10
	2	0.18^*^	0.10^*^	−0.15^*^	0.16^*^	−0.06	−0.08	−0.05	−0.13^*^	−0.10	−0.11^*^
	3	0.27^*^	0.06	−0.22^*^	0.12^*^	−0.14^*^	−0.09	−0.05	−0.12^*^	−0.09	−0.09
	4	0.17^*^	0.02	−0.15^*^	0.06	−0.09	−0.08	−0.01	−0.08	−0.11^*^	−0.05
Number Percentage of Forage Species	1	−0.23^*^	0.00	0.16^*^	−0.04	0.14^*^	0.13^*^	0.00	0.11^*^	0.14^*^	0.12^*^
	2	0.15^*^	−0.11^*^	−0.12^*^	−0.21^*^	−0.15^*^	−0.06	−0.04	−0.01	0.05	0.06
	3	0.12^*^	−0.11^*^	−0.09	−0.07	−0.08	−0.05	0.05	0.01	−0.06	−0.01
	4	0.17^*^	0.15^*^	−0.10^*^	0.16^*^	−0.02	−0.09	−0.05	−0.13^*^	−0.13^*^	−0.14^*^
Biomass of Forage Species	1	−0.25^*^	0.07	0.16^*^	0.11^*^	0.18^*^	0.05	0.05	0.03	0.14^*^	0.08
	2	0.23^*^	−0.15^*^	−0.20^*^	−0.17^*^	−0.22^*^	−0.04	0.05	0.06	0.07	0.07
	3	0.20^*^	−0.17^*^	−0.19^*^	−0.07	−0.19^*^	−0.05	0.04	−0.02	−0.08	0.01
	4	0.24^*^	0.06	−0.21^*^	0.14^*^	−0.12^*^	−0.09	−0.04	−0.13^*^	−0.10	−0.09
Biomass Percentage of Forage Species	1	−0.30^*^	0.13^*^	0.24^*^	0.14^*^	0.24^*^	0.06	0.00	0.01	0.11^*^	0.05
	2	0.23^*^	−0.10	−0.14^*^	−0.17^*^	−0.18^*^	−0.03	0.00	0.04	0.06	0.06
	3	0.19^*^	−0.17^*^	−0.18^*^	−0.06	−0.18^*^	−0.04	0.03	−0.02	−0.07	0.01
	4	0.23^*^	0.06	−0.20^*^	0.13^*^	−0.11^*^	−0.08	−0.04	−0.12^*^	−0.11^*^	−0.10

**Notes.**

* indicates a significant correlation at the *p* < 0.05 level

SBD soil bulk density SOC soil organic carbon GDP gross domestic product

Five sites dominated by *Pennisetum sinese* were excluded from the analysis.

Grazing (185 sites out of all 373 sampling sites) was associated with a significant reduction in the biomass percentage of the preferred forage species (38.5%) when compared with sites characterized by no grazing (46.0%, 188 sites out of all 373 sampling sites). For under grazing, the biomass percentages of desirable forage species (33.8%), consumed but undesirable forage species (5.1%), and non-consumable or toxic forage species (7.3%) were consistently higher than those under no grazing (31.0%, 3.9%, and 6.2%), but not statistically significant.

For plant community characteristics, the number of species tended to be positively correlated with the number and biomass of forage species of different forage quality levels. The correlations tended to be negative for the number percentage and biomass (biomass and biomass percentage) of preferred forage species (Table 3). For plant height, there tended to be a positive correlation between plant height and the number and amount of preferred forage species, but such correlations tended to be negative for other forage species (Table 3). Plant cover was not observed to be significantly correlated with grassland quality levels and was only positively correlated with biomass and biomass percentage of desirable forage species. Biomass tended to be positively correlated with the biomass of different forage quality levels, but not significantly correlated with the number, number percentage, and biomass percentage of species of different forage quality levels (Table 3).

**Table 3:** The correlation coefficients between grassland quality and plant community characteristics.

	**Forage Quality Level**	**Number of species**	**Height**	**Plant cover**	**Total** **Biomass**
Number of Forage Species	1	0.32^*^	−0.05	−0.01	0.04
	2	0.29^*^	0.00	−0.04	0.05
	3	0.36^*^	−0.10	−0.01	−0.01
	4	0.34^*^	−0.05	−0.03	0.08
Number Percentage of Forage Species	1	−0.36^*^	0.17^*^	0.00	0.02
	2	0.17^*^	−0.21^*^	0.08	−0.04
	3	0.10	−0.01	−0.06	0.10^*^
	4	0.20^*^	0.02	−0.03	0.01
Biomass of Forage Species	1	−0.19^*^	0.39^*^	0.02	0.47^*^
	2	0.18^*^	−0.11^*^	0.20^*^	0.30^*^
	3	0.24^*^	−0.08	−0.08	0.14^*^
	4	0.33^*^	−0.04	−0.01	0.07
Biomass Percentage of Forage Species	1	−0.26^*^	0.29^*^	−0.07	0.11^*^
	2	0.13^*^	−0.23^*^	0.13^*^	−0.06
	3	0.24^*^	−0.11^*^	−0.09	0.06
	4	0.32^*^	−0.05	−0.01	0.02

**Notes.**

* indicates a significant correlation at the *p* < 0.05 level.

Five sites dominated by *Pennisetum sinese* were excluded from the analysis.

## Discussion

This study evaluated the forage quality levels of 272 species belonging to 48 families and 177 genera distributed within 373 sampling sites throughout Guizhou Province in the karst mountain region of Southwest China. Although these 272 species account for about 56.3% of all species found (483 species), however, they constituted 85.9% of total biomass. There were 65 preferred forage species, 151 desirable forage species, 33 consumable but undesirable forage species, and 23 non-consumable or toxic forage species. The grassland forage quality information obtained in this study can be used for high quality forage protection and grassland improvement throughout karst mountain regions (Shi et al., 2013; Li et al., 2018a; Li et al., 2018b; Li et al., 2018c; Yang et al., 2022).

**Figure 3:**
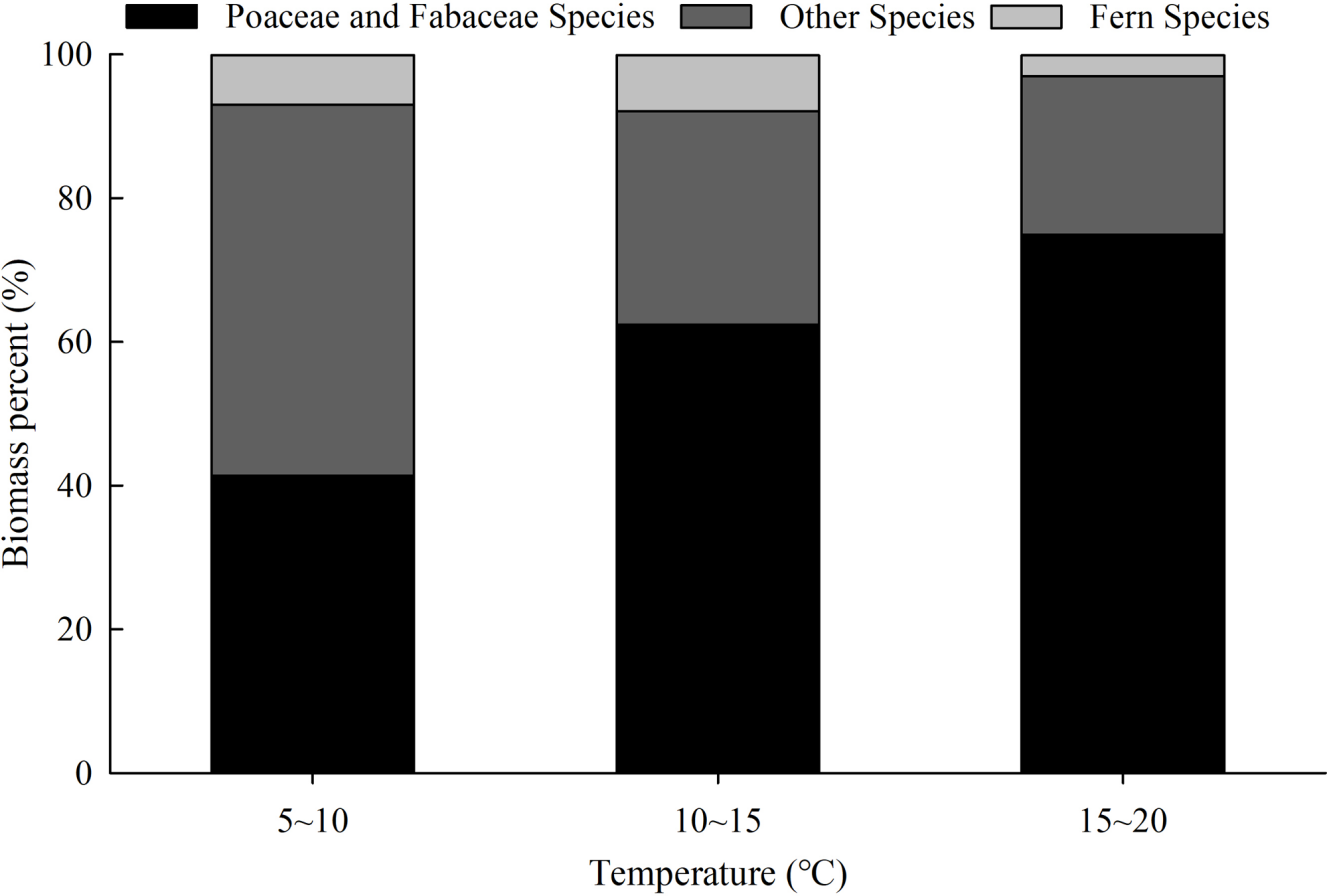
The variation of grassland plant community composition with temperature. Fern species includes species in the families: Thelypteridaceae, Onocleaceae, Pteridaceae, Lindsaeaceae, Osmundaceae, and Blechnaceae.

The anticipated rise in temperatures due to global climate change is likely going to facilitate the growth of preferred forage species but confine the growth of desirable, consumable but undesirable, and non-consumable or toxic forage species. This is consistent with other studies that show that warming can increase rangeland grass quality as well as shift plant community composition on the Tibetan Plateau (Wang et al., 2012; Polley et al., 2013; Li et al., 2018a; Li et al., 2018b; Li et al., 2018c). In this study, the biomass percentage of gramineous and leguminous species (mostly considered to be preferred forage species) increased from about 40% (41.4%) to about 75% (74.9%) when the temperatures increased from 5–10 °C to 15–20 °C (Fig. 3). At the same time, when the temperature was increased from 5–10 °C to 15–20 °C there were sharp decreases in biomass percentages for fern species (including those in the families: Thelypteridaceae, Onocleaceae, Pteridaceae, Lindsaeaceae, Osmundaceae, and Blechnaceae, which are mostly considered to be non-consumable or toxic forage species) and other plants, that dropped from 7.0% to 3.0% and 51.6% to 22.1%, respectively (Fig. 3). Similar to previous studies (Li et al., 2018a; Li et al., 2018b; Li et al., 2018c), increase in precipitation was observed to be positively associated with forage quality and biomass percentages. Meanwhile, higher precipitation can also pose a higher risk of increasing the number and number percentage of non-consumable or toxic forage species (Table 2). Elevation may also be an indirect factor, as there have been correlations observed between elevation and precipitation and temperature in the study area (Deng et al., 2015; Yue et al., 2018). Therefore, we argue that expected climate warming and increasing precipitation may have a positive impact on the grassland quality of the karst mountain region of Southwest China (IPCC, 2022). Additional warming experiments on grassland forage quality and plant community composition are needed in areas such as the karst mountain region of Southwest China.

Soil attributes, including SBD and SOC, do not show a strong correlation with grassland forage quality. This is consistent with previous research showing that soil attributes were not important factors affecting grassland quality, however, soil pH was not considered in this research (Shi et al., 2013). More than 80% (83.7%) of the study area has acidic soil (pH <7.0), and 59.4% of the land surface of the study area have been observed to have pH values that were less than 6.0 (FAO et al., 2012). Soil pH is positively associated with the number and biomass of preferred forage plants but negatively associated with the occurrence of other plant species, especially those that are non-consumable or toxic, indicating soil improvement measures to ameliorate soil acidity may have positive implications on grassland quality. Future research on the specific mechanism for such a positive implication is needed. Given the highly heterogeneous environmental pattern of the karst grasslands, compared with the large-scale soil and climate data used in this paper, more accurate soil probing and climate measurements at the sampling sites likely can provide more detailed information on the interactions between environmental factors and karst grassland forage quality of Southwest China in future studies.

Both GDP and population density were positively correlated with the numbers, percentages and biomass of preferred forage species, but negatively correlated for species at lower levels of forage quality. People living in regions with better economic conditions can invest more in grassland improvement measures such as reseeding and grazing exclusion to enhance the quality of grassland (Shang et al., 2014; Briske et al., 2015). Thus, the rapid economic growth of Guizhou Province and other provinces in the karst mountain region of Southwest China in recent years (National Bureau of Statistics of China, https://data.stats.gov.cn/english/, accessed on August 15, 2021) may facilitate future grassland quality improvement.

Grazing can decrease the abundance of preferred forage species in karst grasslands of Guizhou Province of Southwest China. This observation is consistent with previous studies demonstrating that heavy grazing results in the consumption of high quality forage quality and thus the reduced forage quality of grasslands (Bokdam & De Vries Wallis, 1992; Asner et al., 2004). This finding indicated that from the perspective of maintaining grassland quality, no grazing or low-level grazing is important in the karst mountain region of Southwest China.

There was a negative correlation between plant biodiversity and the amount of preferred forage species, but a positive correlation for non-consumable or toxic forage species. This result is consistent with previous studies that demonstrate that plant diversity is often associated with low biomass and forage quality in agricultural settings (Tallowin & Jefferson, 1999; Bruinenberg et al., 2002; White, Barker & Moore, 2004; Isselstein, Jeangros & Pavlu, 2005). The reported grassland ecosystems in Guizhou Province lie between natural grasslands and complete agricultural settings, and grassland forage quality may be directly affected by plant species composition (French, 2017). These mechanisms underlying the community scale correlations between grassland forage quality and plant community structures are important and merit further exploration.

The results of the present study suggest that the predicted global warming and the rapid economic growth in Guizhou Province may increase grassland forage quality. Therefore, through focusing efforts on grassland and soil improvement, as well as maintaining an appropriate grazing intensity, grassland quality can be improved in the karst mountain region of Southwest China.

##  Supplemental Information

10.7717/peerj.15323/supp-1 Supplemental Information 1 All forage plant species in grasslands of the karst mountain region of Guizhou Province, Southwest China The four quality levels are as follows: preferred (level 1), desirable (level 2), consumed but undesirable (level 3), non-consumable or toxic (level 4).

10.7717/peerj.15323/supp-2 Supplemental Information 2 The forage quality level of each plant species in grasslands of the karst mountain region of Guizhou Province in Southwest China

10.7717/peerj.15323/supp-3 Supplemental Information 3 The data for grassland forage quality and social and environmental factors

## Additional Information and Declarations

### Field Study Permissions

The following information was supplied relating to field study approvals (i.e., approving body and any reference numbers): The sampling sites were selected throughout Guizhou Province under the approval from Guizhou Department of Natural Resources.

### Data Availability

The following information was supplied regarding data availability: The raw data is available in the Supplemental Files.
