# Supplementary material for: Karst grassland forage quality and its determinants in Guizhou Province of Southwest China
Source: PeerJ. 2023 May 16;11:e15323. doi: 10.7717/peerj.15323 (PMC10198152; doi:10.7717/peerj.15323)
Supplement: Supplemental Information 1 — The four quality levels are as follows: preferred (level 1), desirable (level 2), consumed but undesirable (level 3), non-consumable or toxic (level 4). [file peerj-11-15323-s001.docx]

**Appendix Table S1** All forage plant species in grasslands of the karst mountain region of Guizhou ProvinceSouthwest China. The four quality levels are as follows: preferred (level 1), desirable (level 2), consumed but undesirable (level 3), non-consumable or toxic (level 4) (Chen & Jia, 2002; Damiran, 2005).

| **Rank** | **Family** | **Name** | **Forage Quality Level** |
| --- | --- | --- | --- |
| 1 | *Poaceae* | *Imperata cylindrica (L.) P.Beauv.* | 1 |
| 2 | *Poaceae* | *Miscanthus floridulus (Labill.) Warb. ex K.Schum. & Lauterb.* | 1 |
| 3 | *Poaceae* | *Pennisetum sinese Roxb* | 1 |
| 4 | *Poaceae* | *Arthraxon hispidus (Trin.) Makino* | 1 |
| 5 | *Fabaceae* | *Trifolium repens L.* | 1 |
| 6 | *Poaceae* | *Pennisetum alopecuroides (L.) Spreng.* | 1 |
| 7 | *Poaceae* | *Setaria viridis (L.) P.Beauv.* | 1 |
| 8 | *Poaceae* | *Pogonatherum crinitum (Thunb.) Kunth* | 1 |
| 9 | *Poaceae* | *Leptochloa chinensis (L.) Nees* | 1 |
| 10 | *Poaceae* | *Lolium perenne L.* | 1 |
| 11 | *Poaceae* | *Bothriochloa ischaemum (L.) Keng* | 1 |
| 12 | *Fabaceae* | *Kummerowia striata (Thunb.) Schindl.* | 1 |
| 13 | *Cyperaceae* | *Fimbristylis dichotoma (L.) Vahl* | 1 |
| 14 | *Poaceae* | *Dactylis glomerata L.* | 1 |
| 15 | *Poaceae* | *Echinochloa crus-galli (L.) P. Beauv.* | 1 |
| 16 | *Poaceae* | *Cynodon dactylon (L.) Pers.* | 1 |
| 17 | *Fabaceae* | *Dunbaria villosa (Thunb.) Makino* | 1 |
| 18 | *Lamiaceae* | *Origanum vulgare L.* | 1 |
| 19 | *Poaceae* | *Paspalum dilatatum Poir.* | 1 |
| 20 | *Poaceae* | *Bromus catharticus Vahl* | 1 |
| 21 | *Lamiaceae* | *Perilla frutescens (L.) Britton* | 1 |
| 22 | *Fabaceae* | *Flemingia macrophylla (Willd.) O.Ktze. ex Prain* | 1 |
| 23 | *Amaranthaceae* | *Amaranthus hybridus L.* | 1 |
| 24 | *Asteraceae* | *Artemisia stechmanniana Besser* | 1 |
| 25 | *Amaranthaceae* | *Amaranthus cruentus L.* | 1 |
| 26 | *Fabaceae* | *Glycine soja Siebold & Zucc.* | 1 |
| 27 | *Poaceae* | *Leptochloa panicea (Retz.) Ohwi* | 1 |
| 28 | *Poaceae* | *Phragmites australis (Cav.) Trin. ex Steud.* | 1 |
| 29 | *Fabaceae* | *Lespedeza tomentosa (Thunb.) Siebold ex Maxim.* | 1 |
| 30 | *Asteraceae* | *Hemisteptia lyrata (Bunge) Fisch. & C.A.Mey.* | 1 |
| 31 | *Poaceae* | *Setaria palmifolia (J.Koenig) Stapf* | 1 |
| 32 | *Fabaceae* | *Kummerowia stipulacea (Maxim.) Makino* | 1 |
| 33 | *Polygonaceae* | *Polygonum capitatum Buch.-Ham. ex D.Don* | 1 |
| 34 | *Plantaginaceae* | *Plantago asiatica L.* | 1 |
| 35 | *Fabaceae* | *Trifolium pratense L.* | 1 |
| 36 | *Amaryllidaceae* | *Allium ramosum L.* | 1 |
| 37 | *Commelinaceae* | *Commelina communis L.* | 1 |
| 38 | *Poaceae* | *Avena sativa L.* | 1 |
| 39 | *Poaceae* | *Imperata cylindrica var. major (Nees) C. E. Hubbard* | 1 |
| 40 | *Poaceae* | *Eremochloa ophiuroides (Munro) Hack.* | 1 |
| 41 | *Fabaceae* | *Lotus corniculatus L.* | 1 |
| 42 | *Poaceae* | *Lolium multiflorum Lam.* | 1 |
| 43 | *Fabaceae* | *Lespedeza bicolor Turcz.* | 1 |
| 44 | *Fabaceae* | *Vicia sepium L.* | 1 |
| 45 | *Apiaceae* | *Cryptotaenia japonica Hassk.* | 1 |
| 46 | *Fabaceae* | *Desmodium heterocarpon (L.) DC.* | 1 |
| 47 | *Asteraceae* | *Taraxacum mongolicum Hand.-Mazz.* | 1 |
| 48 | *Cyperaceae* | *Fimbristylis littoralis Gaudich.* | 1 |
| 49 | *Poaceae* | *Zoysia japonica Steud.* | 1 |
| 50 | *Poaceae* | *Setaria pumila (Poir.) Roem. & Schult.* | 1 |
| 51 | *Saururaceae* | *Houttuynia cordata Thunb.* | 1 |
| 52 | *Fabaceae* | *Medicago sativa L.* | 1 |
| 53 | *Convolvulaceae* | *Calystegia hederacea Wall.* | 1 |
| 54 | *Portulacaceae* | *Portulaca oleracea L.* | 1 |
| 55 | *Poaceae* | *Leymus chinensis (Trin.) Tzvelev* | 1 |
| 56 | *Polygonaceae* | *Polygonum nepalense Meisn.* | 1 |
| 57 | *Polygonaceae* | *Polygonum chinense L.* | 1 |
| 58 | *Poaceae* | *Eragrostis minor Host* | 1 |
| 59 | *Poaceae* | *Arthraxon prionodes (Steud.) Dandy* | 1 |
| 60 | *Polygonaceae* | *Polygonum perfoliatum (L.) L.* | 1 |
| 61 | *Moraceae* | *Broussonetia papyrifera (L.) Vent.* | 1 |
| 62 | *Fabaceae* | *Albizia julibrissin Durazz.* | 1 |
| 63 | *Asteraceae* | *Taraxacum sinicum Kitag.* | 1 |
| 64 | *Fabaceae* | *Cajanus cajan (L.) Millsp.* | 1 |
| 65 | *Asteraceae* | *Lactuca indica L.* | 1 |
| 66 | *Poaceae* | *Eragrostis pilosa (L.) P.Beauv.* | 2 |
| 67 | *Poaceae* | *Arundinella hirta (Thunb.) Tanaka* | 2 |
| 68 | *Poaceae* | *Heteropogon contortus (L.) P.Beauv. ex Roem. & Schult.* | 2 |
| 69 | *Poaceae* | *Pennisetum purpureum Schumach.* | 2 |
| 70 | *Asteraceae* | *Artemisia argyi H.Lév. & Vaniot* | 2 |
| 71 | *Poaceae* | *Cymbopogon goeringii (Steud.) A.Camus* | 2 |
| 72 | *Poaceae* | *Cymbopogon mekongensis A.Camus* | 2 |
| 73 | *Poaceae* | *Paspalum thunbergii Kunth ex Steud.* | 2 |
| 74 | *Poaceae* | *Digitaria sanguinalis (L.) Scop.* | 2 |
| 75 | *Poaceae* | *Calamagrostis pseudophragmites (Haller f.) Koeler* | 2 |
| 76 | *Poaceae* | *Paspalum distichum L.* | 2 |
| 77 | *Cyperaceae* | *Cyperus rotundus L.* | 2 |
| 78 | *Poaceae* | *Capillipedium parviflorum (R.Br.) Stapf* | 2 |
| 79 | *Rosaceae* | *Potentilla chinensis Ser.* | 2 |
| 80 | *Asteraceae* | *Erigeron acris L.* | 2 |
| 81 | *Asteraceae* | *Artemisia sieversiana Ehrh. ex Willd.* | 2 |
| 82 | *Poaceae* | *Calamagrostis epigeios (L.) Roth* | 2 |
| 83 | *Rosaceae* | *Agrimonia pilosa Ledeb.* | 2 |
| 84 | *Urticaceae* | *Gonostegia hirta (Blume) Miq.* | 2 |
| 85 | *Asteraceae* | *Artemisia selengensis Turcz. ex Besser* | 2 |
| 86 | *Polygonaceae* | *Polygonum hydropiper L.* | 2 |
| 87 | *Asteraceae* | *Artemisia dubia Wall. ex Besser* | 2 |
| 88 | *Poaceae* | *Pennisetum flaccidum Griseb.* | 2 |
| 89 | *Rosaceae* | *Duchesnea indica (Andr.) Focke* | 2 |
| 90 | *Cyperaceae* | *Carex cruciata Wahlenb.* | 2 |
| 91 | *Poaceae* | *Miscanthus sinensis Andersson* | 2 |
| 92 | *Poaceae* | *Tripogon chinensis (Franch.) Hack.* | 2 |
| 93 | *Poaceae* | *Apluda mutica L.* | 2 |
| 94 | *Poaceae* | *Eragrostis ferruginea (Thunb.) P.Beauv.* | 2 |
| 95 | *Poaceae* | *Sporobolus fertilis (Steud.) Clayton* | 2 |
| 96 | *Juncaceae* | *Juncus effusus L.* | 2 |
| 97 | *Asteraceae* | *Erigeron annuus (L.) Pers.* | 2 |
| 98 | *Poaceae* | *Stipa capillata L.* | 2 |
| 99 | *Poaceae* | *Themeda triandra Forssk.* | 2 |
| 100 | *Asteraceae* | *Carpesium cernuum L.* | 2 |
| 101 | *Urticaceae* | *Debregeasia orientalis C.J.Chen* | 2 |
| 102 | *Fabaceae* | *Indigofera amblyantha Craib* | 2 |
| 103 | *Poaceae* | *Eleusine indica (L.) Gaertn.* | 2 |
| 104 | *Poaceae* | *Echinochloa crus-galli var. mitis (Pursh) Petermann* | 2 |
| 105 | *Polygonaceae* | *Polygonum divaricatum L.* | 2 |
| 106 | *Cyperaceae* | *Carex breviculmis R.Br.* | 2 |
| 107 | *Cyperaceae* | *Eriophorum comosum (Wall.) Nees* | 2 |
| 108 | *Apiaceae* | *Hydrocotyle sibthorpioides Lam.* | 2 |
| 109 | *Plantaginaceae* | *Plantago depressa Willd.* | 2 |
| 110 | *Gentianaceae* | *Swertia bimaculata (Siebold & Zucc.) Hook.fil. & Thomson ex C.B.Clarke* | 2 |
| 111 | *Anacardiaceae* | *Rhus chinensis Mill.* | 2 |
| 112 | *Polygonaceae* | *Rumex acetosa L.* | 2 |
| 113 | *Poaceae* | *Festuca glauca Vill.* | 2 |
| 114 | *Fabaceae* | *Pueraria montana (Lour.) Merr.* | 2 |
| 115 | *Poaceae* | *Saccharum rufipilum Steud.* | 2 |
| 116 | *Cyperaceae* | *Cyperus iria L.* | 2 |
| 117 | *Lycopodiaceae* | *Stellaria media (L.) Vill.* | 2 |
| 118 | *Urticaceae* | *Boehmeria nivea (L.) Gaudich.* | 2 |
| 119 | *Amaranthaceae* | *Alternanthera philoxeroides (Mart.) Griseb.* | 2 |
| 120 | *Fabaceae* | *Desmodium sequax Wall.* | 2 |
| 121 | *Equisetaceae* | *Equisetum hyemale L.* | 2 |
| 122 | *Poaceae* | *Panicum sumatrense Roth ex Roem. & Schult.* | 2 |
| 123 | *Malvaceae* | *Urena lobata L.* | 2 |
| 124 | *Brassicaceae* | *Lepidium apetalum Willd.* | 2 |
| 125 | *Rosaceae* | *Geum aleppicum Jacq.* | 2 |
| 126 | *Fabaceae* | *Lespedeza chinensis G. Don* | 2 |
| 127 | *Asteraceae* | *Artemisia caruifolia Roxb.* | 2 |
| 128 | *Gentianaceae* | *Gentiana scabra Bunge* | 2 |
| 129 | *Rosaceae* | *Rubus parkeri Hance* | 2 |
| 130 | *Rosaceae* | *Geum japonicum var. chinense F.Bolle* | 2 |
| 131 | *Apiaceae* | *Centella asiatica (L.) Urb.* | 2 |
| 132 | *Fabaceae* | *Indigofera tinctoria L.* | 2 |
| 133 | *Poaceae* | *Capillipedium assimile (Steud.) A.Camus* | 2 |
| 134 | *Fabaceae* | *Sophora davidii (Franch.) Skeels* | 2 |
| 135 | *Cyperaceae* | *Carex neurocarpa Maxim.* | 2 |
| 136 | *Lamiaceae* | *Perilla frutescens var. purpurascens (Hayata) H.W.Li* | 2 |
| 137 | *Fabaceae* | *Uraria lagopodioides (L.) DC.* | 2 |
| 138 | *Cyperaceae* | *Carex liparocarpos Gaudin* | 2 |
| 139 | *Fabaceae* | *Rhynchosia volubilis Lour.* | 2 |
| 140 | *Poaceae* | *Avena fatua L.* | 2 |
| 141 | *Primulaceae* | *Lysimachia fortunei Maxim.* | 2 |
| 142 | *Lamiaceae* | *Leonurus japonicus Houtt.* | 2 |
| 143 | *Poaceae* | *Echinochloa crus-galli var. zelayensis (Kunth) Hitchc.* | 2 |
| 144 | *Fabaceae* | *Lespedeza juncea (L. f.) Pers.* | 2 |
| 145 | *Lygodiaceae* | *Lygodium japonicum (Thunb.) Sw.* | 2 |
| 146 | *Asteraceae* | *Sigesbeckia orientalis L.* | 2 |
| 147 | *Fabaceae* | *Pueraria peduncularis (Benth.) Graham ex Benth.* | 2 |
| 148 | *Asteraceae* | *Galinsoga parviflora Cav.* | 2 |
| 149 | *Fabaceae* | *Styphnolobium japonicum (L.) Schott* | 2 |
| 150 | *Fabaceae* | *Lespedeza davidii Franch.* | 2 |
| 151 | *Gentianaceae* | *Halenia elliptica D.Don* | 2 |
| 152 | *Polygonaceae* | *Polygonum paleaceum Wall. ex Hook.fil.* | 2 |
| 153 | *Euphorbiaceae* | *Acalypha australis L.* | 2 |
| 154 | *Fabaceae* | *Desmodium gangeticum (L.) DC.* | 2 |
| 155 | *Plantaginaceae* | *Veronica persica Poir.* | 2 |
| 156 | *Fabaceae* | *Lespedeza cuneata (Dum.Cours.) G. Don* | 2 |
| 157 | *Asteraceae* | *Youngia japonica (L.) DC.* | 2 |
| 158 | *Gentianaceae* | *Gentiana macrophylla Pall.* | 2 |
| 159 | *Salicaceae* | *Populus adenopoda Maxim.* | 2 |
| 160 | *Polygonaceae* | *Fagopyrum dibotrys (D.Don) Hara* | 2 |
| 161 | *Fabaceae* | *Lespedeza pilosa (Thunb.) Siebold & Zucc.* | 2 |
| 162 | *Oxalidaceae* | *Oxalis corniculata L.* | 2 |
| 163 | *Asteraceae* | *Sonchus oleraceus L.* | 2 |
| 164 | *Poaceae* | *Axonopus compressus (Sw.) Beauv.* | 2 |
| 165 | *Fabaceae* | *Campylotropis polyantha (Franch.) Schindl.* | 2 |
| 166 | *Asteraceae* | *Lactuca sibirica (L.) Maxim.* | 2 |
| 167 | *Amaranthaceae* | *Achyranthes bidentata Blume* | 2 |
| 168 | *Asteraceae* | *Ixeris polycephala Cass.* | 2 |
| 169 | *Asteraceae* | *Anaphalis flavescens Hand.-Mazz.* | 2 |
| 170 | *Primulaceae* | *Lysimachia christiniae Hance* | 2 |
| 171 | *Asteraceae* | *Crassocephalum crepidioides (Benth.) S.Moore* | 2 |
| 172 | *Melastomataceae* | *Melastoma malabathricum L.* | 2 |
| 173 | *Lamiaceae* | *Clerodendrum bungei Steud.* | 2 |
| 174 | *Asteraceae* | *Achillea wilsoniana (Heimerl ex Hand.-Mazz.) Heimerl* | 2 |
| 175 | *Asteraceae* | *Artemisia scoparia Waldst. & Kit.* | 2 |
| 176 | *Poaceae* | *Setaria faberi R.A.W.Herrm.* | 2 |
| 177 | *Asteraceae* | *Emilia sonchifolia (L.) DC.* | 2 |
| 178 | *Fabaceae* | *Cajanus scarabaeoides (L.) Thouars* | 2 |
| 179 | *Asteraceae* | *Eclipta prostrata (L.) L.* | 2 |
| 180 | *Gentianaceae* | *Halenia corniculata (L.) Cornaz* | 2 |
| 181 | *Cyperaceae* | *Cyperus microiria Steud.* | 2 |
| 182 | *Asteraceae* | *Eupatorium lindleyanum DC.* | 2 |
| 183 | *Fabaceae* | *Caesalpinia decapetala (Roth) Alston* | 2 |
| 184 | *Fabaceae* | *Crotalaria albida B.Heyne ex Roth* | 2 |
| 185 | *Rosaceae* | *Rosa cymosa Tratt.* | 2 |
| 186 | *Ranunculaceae* | *Thalictrum minus L.* | 2 |
| 187 | *Anacardiaceae* | *Toxicodendron vernicifluum (Stokes) F.A.Barkley* | 2 |
| 188 | *Amaranthaceae* | *Chenopodium album L.* | 2 |
| 189 | *Equisetaceae* | *Equisetum arvense L.* | 2 |
| 190 | *Asteraceae* | *Sigesbeckia pubescens (Makino) Makino* | 2 |
| 191 | *Euphorbiaceae* | *Triadica sebifera (L.) Small* | 2 |
| 192 | *Fabaceae* | *Parochetus communis Buch.-Ham. ex D.Don* | 2 |
| 193 | *Ranunculaceae* | *Aquilegia viridiflora Pall.* | 2 |
| 194 | *Lygodiaceae* | *Lygodium microphyllum (Cav.) R.Br.* | 2 |
| 195 | *Apiaceae* | *Sanicula chinensis Bunge* | 2 |
| 196 | *Plantaginaceae* | *Plantago major L.* | 2 |
| 197 | *Fabaceae* | *Campylotropis trigonoclada (Franch.) Schindl.* | 2 |
| 198 | *Fabaceae* | *Desmodium microphyllum (Thunb.) DC.* | 2 |
| 199 | *Asteraceae* | *Chrysanthemum indicum L.* | 2 |
| 200 | *Polygalaceae* | *Polygala sibirica L.* | 2 |
| 201 | *Amaranthaceae* | *Amaranthus spinosus L.* | 2 |
| 202 | *Commelinaceae* | *Commelina benghalensis L.* | 2 |
| 203 | *Asteraceae* | *Carpesium abrotanoides L.* | 2 |
| 204 | *Salicaceae* | *Populus yunnanensis Dode* | 2 |
| 205 | *Fabaceae* | *Dalbergia hupeana Hance* | 2 |
| 206 | *Poaceae* | *Poa annua L.* | 2 |
| 207 | *Pinaceae* | *Pinus massoniana Lamb.* | 2 |
| 208 | *Asteraceae* | *Cirsium arvense (L.) Scop.* | 2 |
| 209 | *Rosaceae* | *Potentilla discolor Bunge* | 2 |
| 210 | *Fabaceae* | *Mimosa diplotricha C. Wright* | 2 |
| 211 | *Rosaceae* | *Potentilla kleiniana Wight & Arn.* | 2 |
| 212 | *Fabaceae* | *Bauhinia brachycarpa Wall. ex Benth.* | 2 |
| 213 | *Campanulaceae* | *Adenophora tetraphylla (Thunb.) Fisch.* | 2 |
| 214 | *Asteraceae* | *Eupatorium japonicum Thunb.* | 2 |
| 215 | *Asteraceae* | *Saussurea japonica (Thunb.) DC.* | 2 |
| 216 | *Liliaceae* | *Lilium brownii F.E.Br. ex Miellez* | 2 |
| 217 | *Asteraceae* | *Anaphalis margaritacea (L.) Benth. ex C.B.Clarke* | 3 |
| 218 | *Poaceae* | *Chrysopogon aciculatus (Retz.) Trin.* | 3 |
| 219 | *Asteraceae* | *Bidens pilosa L.* | 3 |
| 220 | *Asteraceae* | *Senecio scandens Buch.-Ham. ex D.Don* | 3 |
| 221 | *Asteraceae* | *Artemisia japonica Thunb.* | 3 |
| 222 | *Plantaginaceae* | *Hippuris vulgaris L.* | 3 |
| 223 | *Poaceae* | *Saccharum arundinaceum Retz.* | 3 |
| 224 | *Asteraceae* | *Inula britannica L.* | 3 |
| 225 | *Asteraceae* | *Anaphalis sinica Hance* | 3 |
| 226 | *Asteraceae* | *Picris hieracioides L.* | 3 |
| 227 | *Asteraceae* | *Carduus nutans L.* | 3 |
| 228 | *Lamiaceae* | *Elsholtzia ciliata (Thunb.) Hyl.* | 3 |
| 229 | *Lamiaceae* | *Stachys oblongifolia Wall. ex Benth.* | 3 |
| 230 | *Zygophyllaceae* | *Tribulus terrestris L.* | 3 |
| 231 | *Lamiaceae* | *Elsholtzia argyi H.Lév.* | 3 |
| 232 | *Orobanchaceae* | *Pedicularis resupinata L.* | 3 |
| 233 | *Rosaceae* | *Sanguisorba officinalis L.* | 3 |
| 234 | *Asteraceae* | *Cirsium shansiense Petr.* | 3 |
| 235 | *Asteraceae* | *Leontopodium leontopodioides (Willd.) Beauverd* | 3 |
| 236 | *Polygonaceae* | *Spodiopogon sibiricus Trin.* | 3 |
| 237 | *Caprifoliaceae* | *Patrinia villosa (Thunb.) Juss.* | 3 |
| 238 | *Asteraceae* | *Leontopodium sinense Hemsl.* | 3 |
| 239 | *Asteraceae* | *Saussurea mongolica (Franch.) Franch.* | 3 |
| 240 | *Asteraceae* | *Cirsium japonicum Fisch. ex DC.* | 3 |
| 241 | *Caprifoliaceae* | *Patrinia monandra C.B.Clarke* | 3 |
| 242 | *Asteraceae* | *Crepis tectorum L.* | 3 |
| 243 | *Asteraceae* | *Bidens tripartita L.* | 3 |
| 244 | *Asteraceae* | *Cirsium vlassovianum Fisch. ex DC.* | 3 |
| 245 | *Asteraceae* | *Cirsium leo Nakai & Kitag.* | 3 |
| 246 | *Asteraceae* | *Aster tataricus L.fil.* | 3 |
| 247 | *Polygalaceae* | *Polygala tenuifolia Willd.* | 3 |
| 248 | *Asteraceae* | *Senecio analogus DC.* | 3 |
| 249 | *Asteraceae* | *Synotis cappa (Buch.-Ham. ex D.Don) C.Jeffrey & Y.L.Chen* | 3 |
| 250 | *Thelypteridaceae* | *Macrothelypteris oligophlebia (Baker) Ching* | 4 |
| 251 | *Onocleaceae* | *Matteuccia struthiopteris (L.) Tod.* | 4 |
| 252 | *Thelypteridaceae* | *Cyclosorus acuminatus (Houtt.) Nakai* | 4 |
| 253 | *Gleicheniaceae* | *Dicranopteris pedata (Houtt.) Nakaike* | 4 |
| 254 | *Sapindaceae* | *Dodonaea viscosa (L.) Jacq.* | 4 |
| 255 | *Asteraceae* | *Ageratina adenophora (Spreng.) R.King & H.Rob.* | 4 |
| 256 | *Pteridaceae* | *Adiantum capillus-veneris L.* | 4 |
| 257 | *Coriariaceae* | *Coriaria nepalensis Wall.* | 4 |
| 258 | *Lycopodiaceae* | *Lycopodium japonicum Thunb.* | 4 |
| 259 | *Pteridaceae* | *Eremochloa ciliaris (L.) Merr.* | 4 |
| 260 | *Pteridaceae* | *Onychium siliculosum (Desv.) C. Chr.* | 4 |
| 261 | *Pteridaceae* | *Onychium japonicum (Thunb.) Kunze* | 4 |
| 262 | *Caprifoliaceae* | *Patrinia scabiosifolia Fisch. ex Link* | 4 |
| 263 | *Asparagaceae* | *Polygonatum odoratum (Mill.) Druce* | 4 |
| 264 | *Ranunculaceae* | *Ranunculus japonicus Thunb.* | 4 |
| 265 | *Osmundaceae* | *Osmunda japonica Thunb.* | 4 |
| 266 | *Lindsaeaceae* | *Odontosoria chinensis (L.) J.Sm.* | 4 |
| 267 | *Thelypteridaceae* | *Cyclosorus parasiticus (L.) Farw.* | 4 |
| 268 | *Asteraceae* | *Eupatorium chinense L.* | 4 |
| 269 | *Blechnaceae* | *Cibotium barometz (L.) J.Sm.* | 4 |
| 270 | *Pteridaceae* | *Pteris cretica L.* | 4 |
| 271 | *Blechnaceae* | *Woodwardia japonica (L.fil.) Sm.* | 4 |
| 272 | *Asteraceae* | *Ligularia sibirica (L.) Cass.* | 4 |
| 273 | *Smilacacea* | *Smilax china L.* | Undetermined |
| 274 | *Cupressaceae* | *Platycladus orientalis (L.) Franco* | Undetermined |
| 275 | *Primulaceae* | *Myrsine africana L.* | Undetermined |
| 276 | *Primulaceae* | *Lysimachia clethroides Duby* | Undetermined |
| 277 | *Plantaginaceae* | *Hemiphragma heterophyllum Wall.* | Undetermined |
| 278 | *Lamiaceae* | *Clinopodium megalanthum (Diels) C.Y.Wu & S.J.Hsuan ex H.W.Li* | Undetermined |
| 279 | *Lamiaceae* | *Clinopodium chinense (Benth.) Kuntze* | Undetermined |
| 280 | *Lamiaceae* | *Anisomeles indica (L.) Kuntze* | Undetermined |
| 281 | *Lamiaceae* | *Agastache rugosa (Fisch. & C.A.Mey.) Kuntze* | Undetermined |
| 282 | *Lamiaceae* | *Nepeta cataria L.* | Undetermined |
| 283 | *Lamiaceae* | *Rosmarinus officinalis L.* | Undetermined |
| 284 | *Lamiaceae* | *Melissa axillaris (Benth.) Bakh.f.* | Undetermined |
| 285 | *Lamiaceae* | *Vitex negundo L.* | Undetermined |
| 286 | *Lamiaceae* | *Clerodendranthus spicatus (Thunb.) C.Y.Wu ex H.W.Li* | Undetermined |
| 287 | *Lamiaceae* | *Mosla scabra (Thunb.) C.Y.Wu & H.W.Li* | Undetermined |
| 288 | *Lamiaceae* | *Mosla chinensis Maxim.* | Undetermined |
| 289 | *Lamiaceae* | *Mosla dianthera (Buch.-Ham. ex Roxb.) Maxim.* | Undetermined |
| 290 | *Lamiaceae* | *Salvia plebeia R.Br.* | Undetermined |
| 291 | *Lamiaceae* | *Prunella vulgaris L.* | Undetermined |
| 292 | *Lamiaceae* | *Isodon rubescens (Hemsl.) H.Hara* | Undetermined |
| 293 | *Lamiaceae* | *Teucrium pernyi Franch.* | Undetermined |
| 294 | *Lamiaceae* | *Elsholtzia splendens Nakai ex F.Maek.* | Undetermined |
| 295 | *Lamiaceae* | *Elsholtzia rugulosa Hemsl.* | Undetermined |
| 296 | *Lamiaceae* | *Caryopteris incana (Thunb. ex Houtt.) Miq.* | Undetermined |
| 297 | *Euphorbiaceae* | *Euphorbia dentata var. rigida Engelm. ex Torr.* | Undetermined |
| 298 | *Euphorbiaceae* | *Vernicia fordii (Hemsl.) Airy Shaw* | Undetermined |
| 299 | *Cannabaceae* | *Humulus scandens (Lour.) Merr.* | Undetermined |
| 300 | *Ranunculaceae* | *Clematis hexapetala Pall.* | Undetermined |
| 301 | *Fabaceae* | *Chamaecrista mimosoides (L.) Greene* | Undetermined |
| 302 | *Fabaceae* | *Hylodesmum podocarpum (DC.) H.Ohashi & R.R.Mill* | Undetermined |
| 303 | *Fabaceae* | *Hylodesmum podocarpum subsp. oxyphyllum* | Undetermined |
| 304 | *Ericaceae* | *Rhododendron simsii Planch.* | Undetermined |
| 305 | *Ericaceae* | *Vaccinium mandarinorum Diels* | Undetermined |
| 306 | *Ericaceae* | *Vaccinium vitis-idaea L.* | Undetermined |
| 307 | *Ericaceae* | *Lyonia ovalifolia (Wall.) Drude* | Undetermined |
| 308 | *Poaceae* | *Phyllostachys edulis (Carrière) J.Houz.* | Undetermined |
| 309 | *Poaceae* | *Neyraudia reynaudiana (Kunth) Keng ex Hitchc.* | Undetermined |
| 310 | *Poaceae* | *Indocalamus tessellatus (Munro) Keng f.* | Undetermined |
| 311 | *Poaceae* | *Sclerochloa dura (L.) P.Beauv.* | Undetermined |
| 312 | *Poaceae* | *Trichurus monsoniae (L.fil.) C.C.Towns.* | Undetermined |
| 313 | *Juglandaceae* | *Platycarya strobilacea Siebold & Zucc.* | Undetermined |
| 314 | *Cucurbitaceae* | *Gynostemma pentaphyllum (Thunb.) Makino* | Undetermined |
| 315 | *Hydrangeaceae* | *Hydrangea chinensis Maxim.* | Undetermined |
| 316 | *Betulaceae* | *Betula luminifera H.J.P.Winkl.* | Undetermined |
| 317 | *Apocynaceae* | *Amsonia tabernaemontana Walter* | Undetermined |
| 318 | *Violaceae* | *Viola betonicifolia Sm.* | Undetermined |
| 319 | *Euphorbiaceae* | *Viola arcuata var. arcuata (Pohl) Müll.Arg.* | Undetermined |
| 320 | *Malvaceae* | *Sida szechuensis Matsuda* | Undetermined |
| 321 | *Malvaceae* | *Hibiscus mutabilis L.* | Undetermined |
| 322 | *Crassulaceae* | *Sedum sarmentosum Bunge* | Undetermined |
| 323 | *Crassulaceae* | *Sedum eythrostictum Miq.* | Undetermined |
| 324 | *Crassulaceae* | *Sedum alfredi Hance* | Undetermined |
| 325 | *Crassulaceae* | *Sedum polytrichoides Hemsl. ex Hemsl. & Forbes* | Undetermined |
| 326 | *Campanulaceae* | *Lobelia davidii Franch.* | Undetermined |
| 327 | *Campanulaceae* | *Codonopsis foetens Hook.f. & Thomson* | Undetermined |
| 328 | *Campanulaceae* | *Campanula pallida Wall.* | Undetermined |
| 329 | *Campanulaceae* | *Adenophora stricta Miq.* | Undetermined |
| 330 | *Campanulaceae* | *Adenophora capillaris Hemsl.* | Undetermined |
| 331 | *Asteraceae* | *Blumea balsamifera (L.) DC.* | Undetermined |
| 332 | *Asteraceae* | *Blumea axillaris (Lam.) DC.* | Undetermined |
| 333 | *Asteraceae* | *Xanthium strumarium L.* | Undetermined |
| 334 | *Asteraceae* | *Chromolaena odorata (L.) R.King & H.Rob.* | Undetermined |
| 335 | *Asteraceae* | *Ageratum conyzoides L.* | Undetermined |
| 336 | *Asteraceae* | *Praxelis clematidea (Griseb.) R.King & H.Rob.* | Undetermined |
| 337 | *Asteraceae* | *Sonchus wightianus DC.* | Undetermined |
| 338 | *Asteraceae* | *Tussilago farfara L.* | Undetermined |
| 339 | *Asteraceae* | *Symphyotrichum subulatum (Michx.) G.L.Nesom* | Undetermined |
| 340 | *Asteraceae* | *Myriactis nepalensis Less.* | Undetermined |
| 341 | *Asteraceae* | *Carpesium scapiform F.H.Chen & C.M.Hu* | Undetermined |
| 342 | *Asteraceae* | *Ainsliaea yunnanensis Franch.* | Undetermined |
| 343 | *Asteraceae* | *Ambrosia artemisiifolia L.* | Undetermined |
| 344 | *Asteraceae* | *Himalaiella deltoidea (Wall. ex DC.) Raab-Straube* | Undetermined |
| 345 | *Asteraceae* | *Inula japonica Thunb.* | Undetermined |
| 346 | *Asteraceae* | *Duhaldea cappa (Buch.-Ham. ex D.Don) Pruski & Anderb.* | Undetermined |
| 347 | *Asteraceae* | *Emilia prenanthoidea DC.* | Undetermined |
| 348 | *Asteraceae* | *Eupatorium fortunei Turcz.* | Undetermined |
| 349 | *Asteraceae* | *Aster baccharoides (Benth.) Steetz* | Undetermined |
| 350 | *Asteraceae* | *Aster hispidus Lam.* | Undetermined |
| 351 | *Asteraceae* | *Aster indicus B.Heyne* | Undetermined |
| 352 | *Selaginellaceae* | *Selaginella uncinata B.Heyne* | Undetermined |
| 353 | *Selaginellaceae* | *Selaginella tamariscina (P.Beauv.) Spring* | Undetermined |
| 354 | *Acanthaceae* | *Barleria cristata L.* | Undetermined |
| 355 | *Acanthaceae* | *Justicia procumbens L.* | Undetermined |
| 356 | *Fagaceae* | *Quercus fabri Hance* | Undetermined |
| 357 | *Fagaceae* | *Castanea seguinii Dode* | Undetermined |
| 358 | *Orchidaceae* | *Spiranthes sinensis (Pers.) Ames* | Undetermined |
| 359 | *Polygonaceae* | *Fallopia multiflora (Thunb.) Haraldson* | Undetermined |
| 360 | *Polygonaceae* | *Polygonum senticosum (Meisn.) Franch. & Sav.* | Undetermined |
| 361 | *Polygonaceae* | *Polygonum posumbu Buch.-Ham.* | Undetermined |
| 362 | *Polygonaceae* | *Polygonum viscosum Buch.-Ham.* | Undetermined |
| 363 | *Polygonaceae* | *Polygonum runcinatum Buch.-Ham.* | Undetermined |
| 364 | *Polygonaceae* | *Rumex hastatus D.Don* | Undetermined |
| 365 | *Orobanchaceae* | *Pedicularis davidii Franch.* | Undetermined |
| 366 | *Onagraceae* | *Epilobium hirsutum L.* | Undetermined |
| 367 | *Onagraceae* | *Oenothera rosea Aiton* | Undetermined |
| 368 | *Onagraceae* | *Oenothera speciosa Nutt.* | Undetermined |
| 369 | *Gentianaceae* | *Gentiana davidi Franch.* | Undetermined |
| 370 | *Gentianaceae* | *Tripterospermum chinense (Migo) Harry Sm.* | Undetermined |
| 371 | *Asclepiadaceae* | *Cynanchum atratum Bunge* | Undetermined |
| 372 | *Verbenaceae* | *Verbena bonariensis L.* | Undetermined |
| 373 | *Verbenaceae* | *Verbena officinalis L.* | Undetermined |
| 374 | *Lamiaceae* | *Callicarpa macrophylla Vahl* | Undetermined |
| 375 | *Aristolochiaceae* | *Asarum heterotropoides F.Schmidt* | Undetermined |
| 376 | *Geraniaceae* | *Geranium dahuricum DC.* | Undetermined |
| 377 | *Geraniaceae* | *Geranium wilfordii Maxim.* | Undetermined |
| 378 | *Geraniaceae* | *Geranium nepalense Sweet* | Undetermined |
| 379 | *Ranunculaceae* | *Thalictrum aquilegiifolium L.* | Undetermined |
| 380 | *Ranunculaceae* | *Clematis ranunculoides Franch.* | Undetermined |
| 381 | *Ranunculaceae* | *Clematis florida Thunb.* | Undetermined |
| 382 | *Ranunculaceae* | *Anemone hupehensis (Hort.) hort.* | Undetermined |
| 383 | *Ranunculaceae* | *Anemone rivularis Buch.-Ham.* | Undetermined |
| 384 | *Ranunculaceae* | *Anemone vitifolia Buch.-Ham. ex DC.* | Undetermined |
| 385 | *Actinidiaceae* | *Actinidia chinensis Planch.* | Undetermined |
| 386 | *Linderniaceae* | *Torenia concolor Lindl.* | Undetermined |
| 387 | *Lardizabalaceae* | *Sargentodoxa cuneata (Oliv.) Rehder & Wilson* | Undetermined |
| 388 | *Lardizabalaceae* | *Akebia trifoliata (Thunb.) Koidz.* | Undetermined |
| 389 | *Oleaceae* | *Forsythia suspensa (Thunb.) Vahl* | Undetermined |
| 390 | *Equisetaceae* | *Equisetum ramosissimum subsp. Debile* | Undetermined |
| 391 | *Equisetaceae* | *Equisetum ramosissimum Desf.* | Undetermined |
| 392 | *Vitaceae* | *Parthenocissus tricuspidata (Siebold & Zucc.) Planch.* | Undetermined |
| 393 | *Vitaceae* | *Ampelopsis glandulosa (Wall.) Momiy.* | Undetermined |
| 394 | *Vitaceae* | *Ampelopsis delavayana Planch. ex Franch.* | Undetermined |
| 395 | *Rubiaceae* | *Serissa serissoides (DC.) Druce* | Undetermined |
| 396 | *Rubiaceae* | *Hedyotis auricularia L.* | Undetermined |
| 397 | *Rubiaceae* | *Hedyotis uncinella Hook. & Arn.* | Undetermined |
| 398 | *Rubiaceae* | *Galium tenuissimum M.Bieb.* | Undetermined |
| 399 | *Rubiaceae* | *Galium spurium L.* | Undetermined |
| 400 | *Rubiaceae* | *Neanotis hirsuta (L.f.) W.H.Lewis* | Undetermined |
| 401 | *Rubiaceae* | *Leptodermis pilosa Diels* | Undetermined |
| 402 | *Rosaceae* | *Pyracantha fortuneana (Maxim.) H.L.Li* | Undetermined |
| 403 | *Rosaceae* | *Malus sieboldii (Regel) Rehder* | Undetermined |
| 404 | *Rosaceae* | *Rosa omeiensis Rolfe* | Undetermined |
| 405 | *Rosaceae* | *Rosa roxburghii Tratt.* | Undetermined |
| 406 | *Rosaceae* | *Rosa multiflora Wrede ex Rössig* | Undetermined |
| 407 | *Rosaceae* | *Spiraea japonica Raf.* | Undetermined |
| 408 | *Rosaceae* | *Spiraea ×bumalda Burv.* | Undetermined |
| 409 | *Rosaceae* | *Spiraea blumei G.Don* | Undetermined |
| 410 | *Rosaceae* | *Spiraea salicifolia L.* | Undetermined |
| 411 | *Rosaceae* | *Rubus innominatus S.Moore* | Undetermined |
| 412 | *Rosaceae* | *Rubus coreanus Miq.* | Undetermined |
| 413 | *Rosaceae* | *Rubus setchuenensis Bureau & Franch.* | Undetermined |
| 414 | *Rosaceae* | *Rubus alceifolius Poir.* | Undetermined |
| 415 | *Rosaceae* | *Rubus lambertianus Ser.* | Undetermined |
| 416 | *Rosaceae* | *Rubus rosifolius Sm.* | Undetermined |
| 417 | *Rosaceae* | *Rubus fockeanus Kurz* | Undetermined |
| 418 | *Rosaceae* | *Rubus parvifolius L.* | Undetermined |
| 419 | *Rosaceae* | *Rubus corchorifolius L.fil.* | Undetermined |
| 420 | *Rosaceae* | *Rubus ellipticus Sm.* | Undetermined |
| 421 | *Rosaceae* | *Cotoneaster horizontalis Decne.* | Undetermined |
| 422 | *Rosaceae* | *Sorbaria arborea C.K.Schneid.* | Undetermined |
| 423 | *Solanaceae* | *Solanum lyratum Thunb.* | Undetermined |
| 424 | *Solanaceae* | *Solanum quitoense Lam.* | Undetermined |
| 425 | *Solanaceae* | *Solanum aculeatissimum Franco & Brea* | Undetermined |
| 426 | *Caprifoliaceae* | *Dipsacus asper Wall.* | Undetermined |
| 427 | *Viburnaceae* | *Viburnum utile Hemsl. ex Forbes & Hemsl.* | Undetermined |
| 428 | *Caprifoliaceae* | *Lonicera japonica Thunb.* | Undetermined |
| 429 | *Apiaceae* | *Cicuta virosa L.* | Undetermined |
| 430 | *Apiaceae* | *Peucedanum praeruptorum Dunn* | Undetermined |
| 431 | *Apiaceae* | *Torilis scabra (Thunb.) DC.* | Undetermined |
| 432 | *Apiaceae* | *Cnidium monnieri (L.) Cusson* | Undetermined |
| 433 | *Moraceae* | *Broussonetia kaempferi Siebold* | Undetermined |
| 434 | *Moraceae* | *Ficus tikoua Bureau* | Undetermined |
| 435 | *Cyperaceae* | *Eleocharis yokoscensis (Franch. & Sav.) Tang & F.T.Wang* | Undetermined |
| 436 | *Cyperaceae* | *Pycreus flavidus (Retz.) T.Koyama* | Undetermined |
| 437 | *Cyperaceae* | *Cyperus cyperoides (L.) Kuntze* | Undetermined |
| 438 | *Cyperaceae* | *Kyllinga polyphylla Willd. ex Link* | Undetermined |
| 439 | *Cyperaceae* | *Carex gibba Wahlenb.* | Undetermined |
| 440 | *Phytolaccaceae* | *Phytolacca acinosa Roxb.* | Undetermined |
| 441 | *Caryophyllaceae* | *Cerastium fontanum Baumg.* | Undetermined |
| 442 | *Caryophyllaceae* | *Gypsophila paniculata L.* | Undetermined |
| 443 | *Iteaceae* | *Itea yunnanensis Franch.* | Undetermined |
| 444 | *Rhamnaceae* | *Rhamnus crenata Siebold & Zucc.* | Undetermined |
| 445 | *Rhamnaceae* | *Berchemia polyphylla Wall.* | Undetermined |
| 446 | *Rhamnaceae* | *Berchemia sinica C.K.Schneid.* | Undetermined |
| 447 | *Rhamnaceae* | *Berchemia kulingensis C.K.Schneid.* | Undetermined |
| 448 | *Rhamnaceae* | *Berchemia lineata (Roem. & Schult.) Benth.* | Undetermined |
| 449 | *Dioscoreaceae* | *Dioscorea alata L.* | Undetermined |
| 450 | *Dioscoreaceae* | *Dioscorea polystachya Turcz.* | Undetermined |
| 451 | *Pinaceae* | *Abies holophylla Maxim.* | Undetermined |
| 452 | *Hypericaceae* | *Hypericum japonicum Thunb.* | Undetermined |
| 453 | *Hypericaceae* | *Hypericum patulum Thunb.* | Undetermined |
| 454 | *Hypericaceae* | *Hypericum monogynum L.* | Undetermined |
| 455 | *Hypericaceae* | *Hypericum seniawinii Maxim.* | Undetermined |
| 456 | *Araliaceae* | *Kalopanax septemlobus (Thunb.) Koidz.* | Undetermined |
| 457 | *Amaranthaceae* | *Suaeda glauca (Bunge) Bunge* | Undetermined |
| 458 | *Amaranthaceae* | *Gomphrena celosioides C.Mart.* | Undetermined |
| 459 | *Berberidaceae* | *Berberis wilsoniae Hemsl.* | Undetermined |
| 460 | *Rehmanniaceae* | *Rehmannia glutinosa (Gaertn.) Libosch. ex Fisch. & C.A.Mey.* | Undetermined |
| 461 | *Linderniaceae* | *Lindernia procumbens (Krock.) Philcox* | Undetermined |
| 462 | *Linderniaceae* | *Lindernia crustacea (L.) F.Muell.* | Undetermined |
| 463 | *Mazaceae* | *Lancea tibetica Hook.fil. & Thomson* | Undetermined |
| 464 | *Mazaceae* | *Mazus miquelii Makino* | Undetermined |
| 465 | *Mazaceae* | *Mazus caducifer Hance* | Undetermined |
| 466 | *Orobanchaceae* | *Siphonostegia chinensis Benth.* | Undetermined |
| 467 | *Scrophulariaceae* | *Buddleja fallowiana Balf.fil. & W.W.Sm.* | Undetermined |
| 468 | *Scrophulariaceae* | *Buddleja lindleyana Fortune ex Lindl.* | Undetermined |
| 469 | *Urticaceae* | *Pilea notata C.H.Wright* | Undetermined |
| 470 | *Commelinaceae* | *Cyanotis arachnoidea C.B.Clarke* | Undetermined |
| 471 | *Melastomataceae* | *Osbeckia stellata Buch.-Ham. ex KerGawl.* | Undetermined |
| 472 | *Melastomataceae* | *Osbeckia chinensis L.* | Undetermined |
| 473 | *Melastomataceae* | *Melastoma dodecandrum Lour.* | Undetermined |
| 474 | *Phyllanthaceae* | *Flueggea virosa (Roxb. ex Willd.) Royle* | Undetermined |
| 475 | *Phyllanthaceae* | *Glochidion puberum (L.) Hutch.* | Undetermined |
| 476 | *Phyllanthaceae* | *Phyllanthus virgatus G.Forst.* | Undetermined |
| 477 | *Iridaceae* | *Iris confusa Sealy* | Undetermined |
| 478 | *Rutaceae* | *Zanthoxylum simulans Hance* | Undetermined |
| 479 | *Rutaceae* | *Zanthoxylum armatum DC.* | Undetermined |
| 480 | *Rutaceae* | *Boenninghausenia albiflora (Hook.) Rchb. ex Meisn.* | Undetermined |
| 481 | *Boraginaceae* | *Cynoglossum divaricatum Stephan ex Lehm.* | Undetermined |
| 482 | *Boraginaceae* | *Cynoglossum furcatum Wall. ex Roxb.* | Undetermined |
| 483 | *Boraginaceae* | *Cynoglossum lanceolatum Hochst. ex A.DC.* | Undetermined |
